# Supplementary material for: Chromothripsis during telomere crisis is independent of NHEJ, and consistent with a replicative origin
Source: Genome Res. 2019 May;29(5):737–49. doi: 10.1101/gr.240705.118 (PMC6499312; doi:10.1101/gr.240705.118)
Supplement: Supplemental Material [file supp_gr.240705.118_Supplemental_file_1.zip › contigs/annotated_contigs/DB39/contig.2.DB39_length_655_mean_cov_6.41221374046.docx]

**DB39_length_655_mean_cov_6.41221374046**

```
ATACAATAAACAAATAAATATATAATATGTGCTAAGAAGAAAAATACAGCAAAATAAGGGGACAGAGAATGATAGGTTGATAGGAAATT
 >chrX:68829694-68829957 + E=2e-146
GGCCAAGGAAAGGTTCTCTGATAAGGTGACATCTAAGAAGAGTCCTGGAGGAAGTAAGGGAGAAGCCATGTGGCTGTCTGGGAGAATAG

CATTTCAGGCAGGAGGAAATACAGGTAAGAAGACCCTTAAGTGGGAGCATTCTTTGCAGGTTCAAGGAACAACCAAGAGTCCAAC|CT|

TTTATCACAGGGTGTCACCTATTCTTCTGAGGTCCCATTTATCTTTCCCCAAATTAATTATTCCTCCTAGAGGCCTACTTCCCCCCTCC
>chrX:68831013-68831404 + E=1e-212
CCTCTTCCCTATGAAAAGGGTCTATAAGCTTCTAGATCTCACTGGGTTGGGCATTCACTTTTTTTTCATGTGATGTCCCCATGCATGTA

AAAAAAATGTGTATGCCTGCTATAGTTTGGATGTGGTTTGTTGTCCCTACCAAATCTCATGTTGAAATTTGATCCCCAACATTGGAGGT

GGTGCCTAATGGGAAGTGTGTTAGTTATGAGAGCAGATTCCTTGCGAATAGATTAATGCCCTCCCTGGGGATGAATGCATTTTTGCTCT

GTTAGTTCCCCCCAGAACTGGTTTTTAAAAAGAG
```
